# Supplementary material for: Patterns of Intron Gain and Loss in Fungi
Source: PLoS Biol. 2004 Nov 30;2(12):e422. doi: 10.1371/journal.pbio.0020422 (PMC532390; doi:10.1371/journal.pbio.0020422)
Supplement: Table S1 — Also available at http://genes.mit.edu/NielsenEtAl/. (4.3 MB ZIP). [file pbio.0020422.st001.zip › NielsenEtAl/html/934.html]

AN2298.1.NCU09006.1.MG01669.1.FG05561.1


```
 CLUSTAL W (1.82) Multiple Sequence Alignments - Introns Inserted


Sequence 1: MG01669.1	449 aa
Sequence 2: FG05561.1	569 aa
Sequence 3: AN2298.1	396 aa
Sequence 4: NCU09006.1	464 aa
Alignment Length: 580 aa
Number Identitical Residues: 146 aa
Alignment Score (without introns) 7347


MG01669.1 	-------------------------------------------------MQDSMTAAALP
NCU09006.1	------------------------------------------------------------
FG05561.1 	MDSSNQNQDQPQAAPEAQQLPDTSAQQGMPPMDVGASMNGNGIPHVPNHVLNPMNPEALT
AN2298.1  	------------------------------------------------------------
          	                                                            

MG01669.1 	PLVQP--------LAEEVQQAQT--IQQPDANGDAHQASG-----------ISAD1EIAL
NCU09006.1	--------------------------------MTTTETNG-------------SD1EIAL
FG05561.1 	MLGDPSLMADPSLLGMQMPMADPSFMMQPGMNLSNGQTQGFSLPVVPPPTTVSAE1EVAL
AN2298.1  	--------------------------------MDSQDQS------------ISPD1EIAL
          	                                    : .             :.: *:**

MG01669.1 	YDRQIRLWGMQAQEK~IRSANVLLVTVKALANEIAKNLVLAGINSLTIVDHEVVTAVDFG
NCU09006.1	YDRQIRLWGMKAQEK~IRSANILLITMKALANEIAKNLVLAGIGSLTINDHAVVSEADLG
FG05561.1 	YDRQIRLWGMAAQAK~IQSANILLITIKALANEIAKNLVLAGVGSLTLLDSATVTEADRG
AN2298.1  	YDRQIRLWGVKAQEK2LRSANILIITFKALANEVAKNLVLAGIGSLTIIDDGIVTEEDLG
          	*********: ** * ::***:*::*.******:********:.***: *   *:  * *

MG01669.1 	AQFLLSEDEGHLGMNRAEAASVNLRKLNPRVNVNVDKEDIRTRGPNYFQNFSVVIATDLD
NCU09006.1	AQFFLSAEDGHLGQNRAIAASASLQRLNPRVKVIVDTDDIRTKHSSFYSSFDIIIATDLD
FG05561.1 	AQFLLPDDEDVIGKNRAQVASVALRKLNPRVHVHVDEEGVKTKGPSYFAAYDIVIATDLD
AN2298.1  	AQFLVN--QDCIGQNRAQAAAPAVRAYNKRVKVYADASGISSKPPEFFGQFDLTIATELD
          	***::   :. :* *** .*:  ::  * **:* .* ..: :: ..::  :.: ***:**

MG01669.1 	PDAFNIINLATRIVNKPFYAAGSHGFYGYIFADLIEHVFVIQRDEGNVATKLQPESRTRD
NCU09006.1	ADTLNVINTATRIHGRKFYAAGSHGLYGFLFADLIEHDFIIERQRSNLSTTLGPETKTRS
FG05561.1 	PESFNIINTATRLNCKAFYAAGCHGLYGFIFSDLIEHDYVIKRDLGNVSTTTGPETRTRS
AN2298.1  	FAMYNVINSACRVAGRPFYAAGLHGFYGFVFSDLIEHDFVITREKSNVPSPIN-ETSTRT
          	    *:** * *:  : ***** **:**::*:***** ::* *: .*:.:    *: ** 

MG01669.1 	VIDVQVKKEGGKS--IERVTKRERYSTWTLS--DVAPLPEEYRKSPRRLRAVTPVLSCLR
NCU09006.1	IVSVAPKEPDNKDSDIELVTKRELYSTWLLASSDASLLPQDILSSPRRKRAVTPILSCLR
FG05561.1 	IVDVQTRKEGPKT--IESVTKRELYSTWFLAS-DVAVLPAEYTQSKRRLKSVTPALSCLR
AN2298.1  	ILNITSKKENDKM--IEMVTKREKYHPLLLA--NTSPLPDELTRLPRRRKQVTPLLSCLR
          	::.:  :: . *   ** ***** * .  *:  :.: ** :     ** : *** *****

MG01669.1 	ALFEFQ---QEK-GRLPALNRREDLERFTTLATERHHKLGLPVSTLKSEVLRSFLQNIGS
NCU09006.1	ALWQFTSTFGVPPTGPELLTNHTALAAFTQMCSDKHKALGLPAETLRAELLRSFLQNVGA
FG05561.1 	ALWEFM---QLQNGRVPSN--REDLKMFTQIATQKHKALGLPSETLRPEFLRSFLQNLVS
AN2298.1  	ALWEFQ---KINHGRLPTFS-HQDLESFTKLARDAHQELKLDISTLDSAFLRTFLSNLGS
          	**::*              . :  *  ** :. : *: * *  .** . .**:**.*: :

MG01669.1 	ELAPVTAIIGGQLAQDVINVLGASQAPIQNTLIFDGNTMEANMYPLHPE---------DD
NCU09006.1	ELAPVAAVLGGQLAQDVINVLGQTQQPIQNFVVFSGESMEANVYALHPR---------EG
FG05561.1 	EISPVSAILGGQLAQDVINVLGQTQQPIQNMVVFDGNTMEALMYPLHPEGPLGASLLTDH
AN2298.1  	ELSPVAGFLGGALAQDVMNVLSAREQPLQNLLLVDGERSVAPIYSLHPF----------Y
          	*::**:..:** *****:***.  : *:** ::..*:   * :*.***            

MG01669.1 	QLGSG---QLVFSSPAGVQFAVD----------SSNAMAAGVVPDPS--LMIPPVNMAGP
NCU09006.1	ELGRGLLPVTMGVIEAAAEVLEKEGQTTDLMGLGGQHLGGGQQQQQQ--QQQPSSQMEGV
FG05561.1 	TVPNGGAPMLMGGGMDGLPLGIDPTAMGALPHHNNPIMLAGGMPQTGNFIAMPGGSLADP
AN2298.1  	SMDTP-------NAMAAVPPAPN------------------PIPLNG----HPGIENGQP
          	 :              .     .                             *  .    

MG01669.1 	DIAG--PGPDMTLGQ---------------------------
NCU09006.1	EQQQQQQQQQQQQQQHANGVSTEQFTGAGQQQEQQGSGAGNA
FG05561.1 	TQLNGAPAPQRPIQEQAAQATAEQPVQEAQANLNASETN---
AN2298.1  	SIGNGQPGVGLS------------------------------
          	
```
